# Novel Reconstructive Technique for Lower Eyelid Defects: Marginal Approach for Releasing the Lid with Closure Handling Technique (MARCH Technique)

**DOI:** 10.3390/jcm14030836

**Published:** 2025-01-27

**Authors:** Anna March De Ribot, Santiago Ortiz-Pérez, Francesc March De Ribot

**Affiliations:** 1 Department of Ophthalmology, Whangarei Hospital, Whangarei 0148, New Zealand; 2 Department of Ophthalmology, Virgen de las Nieves Hospital, 18014 Granada, Spain; drsantiagoortiz@gmail.com; 3 Medical School, University of Granada, 18016 Granada, Spain; 4 Granada Granada Vision and Eye Research Team (G-VER-T), Biosanitary Research Institute of Granada (IBS Granada), 18012 Granada, Spain; 5 Oculoplastics and Facial Aesthetics Unit, Vithas Andaluz Ophthalmological Institute, 18008 Granada, Spain; 6 Otago Medical School, University of Otago, Otago 9016, New Zealand; march.professor@gmail.com

**Keywords:** skin cancer, eyelid cancer, eyelid defects, eyelid reconstruction, eyelid surgery

## Abstract

**Background**: This study introduces a novel surgical approach, the Marginal Approach for Releasing the lid with Closure Handling technique (MARCH technique), a single-step sparing tissue technique, for the reconstruction of medium to large full-thickness lower eyelid defects and its outcomes. **Methods**: The research considers a single-centre case series with a description of the MARCH technique. Patients with a full-thickness medium to large lower eyelid defect underwent this technique, which combines inferior cantholysis, splitting of lamellae and island or advancement flaps. Demographic data, lid defect size, histology and postoperative outcomes were collected. **Results**: The surgical procedure was performed in fifteen patients (nine males and six females). The mean age was 73.9 years (range 48–95 years old). Local anaesthesia was used in 86.7% of cases. The mean defect size was 68.7% (range 50% to 79%) of the lid length. All patients presented good results with no significant complications. **Conclusions**: The MARCH technique seems to be an excellent first-line approach in reconstructing medium to large full-thickness lower eyelid defects. Its single-step approach, tissue-sparing and minimally aggressive nature and ability to potentially guide eyelash positioning and restore the lacrimal system with a more anatomical position make it a promising option. Enrolling more patients and a longer follow-up would provide a better assessment.

## 1. Introduction

Skin cancer is the most common malignancy in humans, with an increasing incidence worldwide. Malignant eyelid tumours are particularly challenging due to the complexity of the anatomical area and its close relationship with the eye. These tumours carry the risk of complications from local invasion into the orbit, as well as the potential for surgical iatrogenic morbidity. Unlike other anatomical areas, where surgical concerns may primarily be cosmetic, eyelid surgery has additional significant functional implications, which can ultimately impact vision [1]. Among the different malignant eyelid cancers, certain types have a typical slow-growing pattern and minimal possibilities of local or distant metastases, such as basal cell carcinoma. In contrast, other types have a rapid-growing behaviour with a higher likelihood of invasion and metastases, such as squamous cell carcinoma [2], sebaceous cell carcinoma [3], or Merkel carcinoma [4].

The most widely accepted treatment for the vast majority of eyelid cancers is the complete excision of the lesion, which often represents removing the full thickness of the eyelid. In such cases, reconstructive surgery plays a significant role in restoring both the function and aesthetics of the eyelids. However, for cases of extensive orbital invasion, more radical procedures, like exenterations, may be required [1], which may imply profound functional and psychological challenges for the patient. The risk of recurrence, more likely in undifferentiated histological subtypes, in large periocular tumours [2,5], and on location at the medial canthus [5], poses additional challenges. In these cases, recurrent eyelid tumours further complicate the reconstructive surgery.

The reconstruction of full-thickness lower eyelid defects bigger than 25% of the eyelid length may require the use of flaps and grafts [6,7,8,9,10]. Surgical procedures like the Tenzel flap [10], the periosteal flap, the tarso-conjunctival flap, also known as the Hughes technique [10], the Smith-modified Kuhnt–Szymanowski procedure with nasal septal grafts or ear cartilage grafts [1,11] or oral mucosa grafts [1] can give satisfactory cosmetic and functional results. However, these techniques sacrifice healthy tissue that could be extremely useful in future surgeries. To avoid this potential problem, we propose a novel surgical technique; we have named this procedure the Marginal Approach for Releasing the lid with Closure Handling technique (MARCH technique), as it provides an accurate description of the procedure while creating an intentional association with the author’s surname. This method offers a safe, tissue-sparing alternative and can serve as a first-line single-step reconstructive surgery to approach lower lid defects of medium and large size.

## 2. Methods

A case series of patients is presented to illustrate the surgical technique. The patients were recruited from March 2022 to December 2024 and gave oral and written consent to both the surgery and the use of images for publishing and teaching purposes. All surgeries were performed by the same surgeon (AMdR). The study was conducted in strict accordance with the principles outlined in the Declaration of Helsinki. Demographics, tumour size, clinical and histologic feature descriptions and postoperative outcomes, including complications, were collected.

### 2.1. Surgical Technique

**Figure 1:**
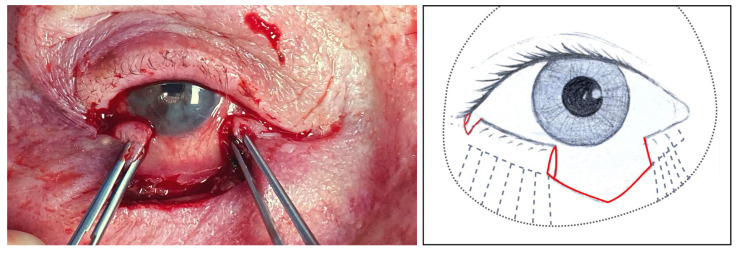
Left, clinical image: The lateral canthal tendon’s inferior crus has been sectioned after tumour removal (Section 3.1.11, patient 11). Right, illustration: The lateral canthus red line represents the incision over the tendon’s inferior crus, the central red line highlights the lid defect after tumour removal, the interrupted grey lines illustrate how the tarsus is attached to the orbital rim, and the black dots represent the orbital rim.

**Figure 2:**
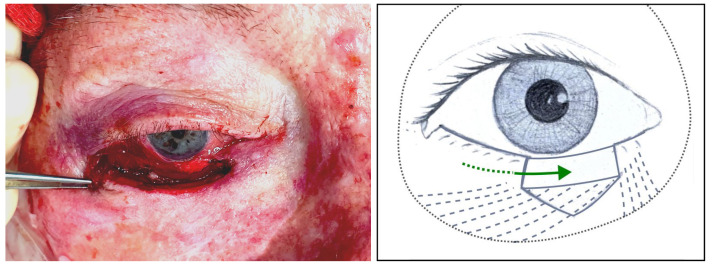
Left, clinical image: Remnant lateral posterior lamella sutured to remnant medial canthus (Section 3.1.11, patient 11). Right, illustration: The green line represents the medial displacement of the posterior lamella, the interrupted grey lines illustrate how the attachment of the tarsus to the orbital rim has changed laterally but persists, and the black dots represent the orbital rim.

**Figure 3:**
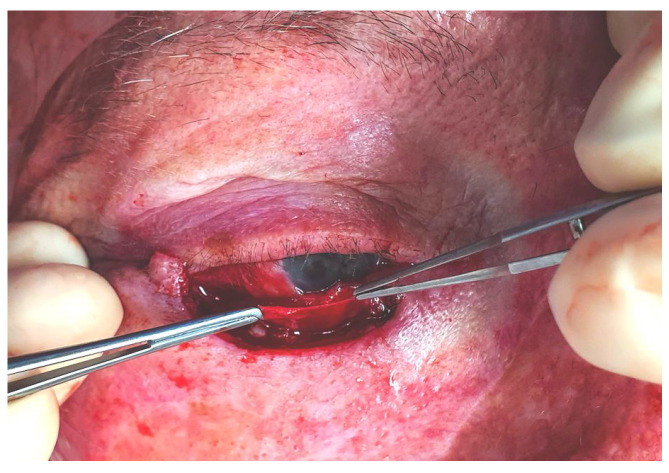
Clinical image: Conjunctiva released from the inferior retractors (Section 3.1.11, patient 11).

**Figure 4:**
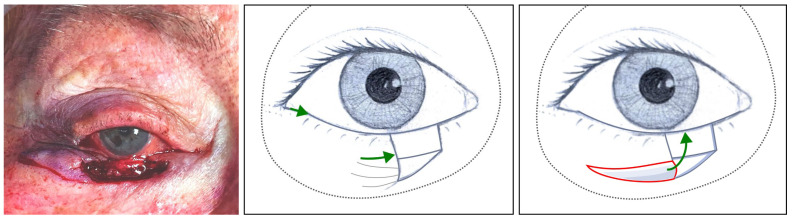
Left, clinical image: Remnant lateral anterior lamella displaced and sutured medially with the incision of the skin for a future island pedicle flap (Section 3.1.11, patient 11). Centre, illustration: The green line represents the medial displacement of the anterior lamella. Right, illustration: The red line describes the limits of the incision to harvest the island pedicle flap, and the green line represents the superior and medial displacement of this flap.

**Figure 5:**
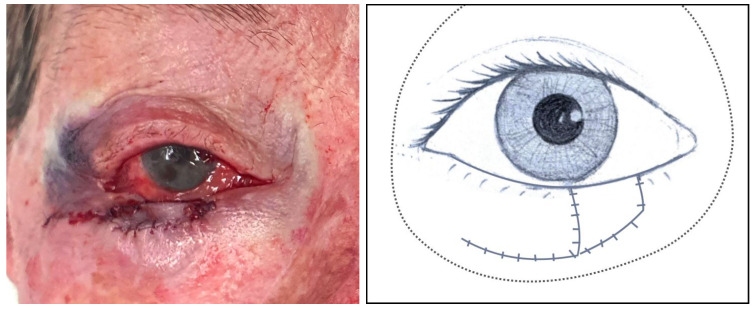
Left, clinical image: Suture of island pedicle flap to complete the reconstruction (Section 3.1.11, patient 11). Right, illustration: Completed suturing.

**Figure 6:**
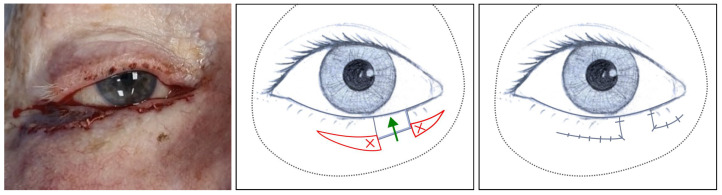
Left, clinical image: The skin incision for an advancement flap and the flap are sutured to complete the reconstruction (Section 3.1.9, patient 9). Centre, illustration: The red line delimits the excess of tissue present, the red crosses represent that this will be removed, and the green line represents the superior displacement of the advancement flap. Right, illustration: Completed suturing.

**Figure 7:**
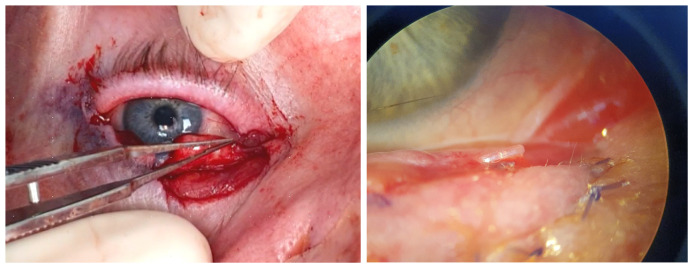
Left, clinical image: Introducing the silicone tube into the remnant inferior canaliculus (Section 3.1.12, patient 12). Right, clinical image: Silicone tube stitched between the remnant posterior lamella and the island pedicle flap (Section 3.1.12, patient 12).

The principle of this technique is based on using the remnant lateral lower eyelid for the whole defect reconstruction. It follows two basic steps: the release of the remnant lateral lower eyelid and the subsequent closure of the lid defect. This can be performed also with a lacrimal system reconstruction when necessary (Figure 1, Figure 2, Figure 3, Figure 4, Figure 5, Figure 6 and Figure 7).

Both the tumour excision and the reconstruction with the MARCH technique can be performed under local anaesthesia (lidocaine 1% and epinephrine 1:200,000, Aspen Pharmacare, St Leonard, Australia), and no special instrumentation, other than a standard 15-blade, Westcott’s scissors (Duckworth & Kent Ltd., Baldock, UK), Castroviejo’s toothed forceps (Duckworth & Kent Ltd., Baldock, UK), Castroviejo needle holder (Katena, Denville, NJ, USA), and 6-0 Vicryl suture (Ethicon, Inc., Cincinnati, OH, USA). The selection and utilisation of alternative surgical instruments or suture materials may be modified according to individual surgeon preferences and expertise.

#### 2.1.1. Step I: Marginal Approach for Releasing the Lid

The surgery starts by sectioning the inferior crus of the lateral canthal tendon to allow the movement of the eyelid and the shortening of the horizontal defect (Figure 1). This is conducted while taking care to damage the skin and the conjunctiva as little as possible. Then, a grey-line incision and splitting of the lamellae of the remnant lateral lower lid are performed. This is an important manoeuvre because it allows the management of the anterior and posterior lamellae separately, as the tension and behaviour of the two lamellae are different. Afterwards, the dissection is extended inferiorly between the orbicularis and the deeper tissues (the tarsal plate and the orbital septum [12]) until the posterior lamella is free and mobile enough to reach the medial end of the defect.

This first part, the release of the lid through the margin, is essential for its posterior closure because of the following: (1) the posterior lamella release allows fixing the medial posterior lamella defect, (2) the dissection running above the septum and the anterior layer of the retractors that remain partially attached to the orbital margin and the medial transposition of the posterior lamella gives it enough tension, and (3) the release of the anterior lamella allows for determining the position of the remnant eyelashes and creating skin folds that benefit the closure of the defect.

#### 2.1.2. Step II: “Closure Handling Technique”

The remnant lateral posterior lamella is sutured to the remnant medial canthus through its lateral tarsal edge if there is any or the medial canthal tendon (Figure 2). Afterwards, its inferior edge is anchored to the conjunctiva, which has previously been released from the inferior retractors (Figure 3).

Then, the remnant lateral anterior lamella is medially displaced and sutured (Figure 4). The decision on the amount of this displacement depends on two main things: (1) whether the quantity of skin folds that are created is enough to allow the whole reconstruction, ensuring the best functional and cosmetic outcome, and (2) where the remnant lateral eyelashes are desired to be placed. If this desired position of the anterior lamella is unreachable with the previous incision and dissection, it may be worth increasing the skin incision laterally and the subcutaneous dissection inferiorly to reach this position.

Finally, to cover the remaining medial defect, the skin folds guide the flap (whether it is an island pedicle flap like in Figure 5 or an advancement flap like in Figure 6). The flap is typically harvested from below and then transposed above the defect, although the approach may vary depending on the case. This individualised strategy is broadly described in the name of this second step as the Closure Handling technique to stress the need for assessment in each patient.

If there is damage to the inferior canaliculus after removing the whole tumour, the canaliculus can be repaired using silicone intubation. The new punctum can be located where it previously was by stitching the tube between the remnant translocated posterior lamella and the advancement or the island pedicle flap (Figure 7). The monocanalicular stent (Mini-Monoka, Paris, France) can be useful in this step.

There are some surgical tips that may be interesting to note. One is to suture the anterior lamella slightly more medially to where you want to leave the remnant eyelashes for the best cosmetic results. Another tip is to consider the additional anchoring sutures to the periosteum for slightly lifting the cheek to prevent an asymmetric lid position if some inferior retraction happens during the scarring process.

Additionally, it is worth mentioning that the difficulty correlates with the size of the defect and the lid laxity, as well as with the size of the remnant lateral lower eyelid.

## 3. Results

Fifteen patients were included in this case series. The 15 patients receiving the MARCH technique for inferior eyelid reconstruction (9 males and 6 females) had a mean age of 73.9 years (range 48–95 years old).

The anaesthesia was local in 86.7% of cases. The other 13.3% required general anaesthesia (Section 3.1.6, Patient 6) and intravenous sedation due to associated anxiety (Section 3.1.7, Patient 7).

The right lower eyelid was involved in 13 patients (86.7% of cases), and the left lower eyelid in 2 patients (13.3% of cases).

The lid defect in all patients was secondary to primary tumour excision. Basal cell carcinoma was the diagnosis in all cases except for Section 3.1.1 (Patient 1). Due to several systemic issues, this patient underwent tumour excision and reconstruction without a confirmed diagnosis. Subsequent pathology analysis revealed a diagnosis of sebaceous hyperplasia.

The mean size of the defect was 18.9 mm (range 15 to 23 mm), accounting for 68.7% (range 50% to 79%) of the lid length. The mean size of the remnant lateral lower lid was 5.6 mm (range 3 to 11 mm), accounting for 20.2% (range 12% to 36.7%) of the lid length. An island flap was used in all cases except for Section 3.1.9 (Patient 9). The mean operative time was 57.5 min (range 35 to 82 min).

Five patients (33.3% of cases) underwent lacrimal system repair during the procedure and a monocanalicular silicone tube was used in all of these cases. No additional processes, such as cheek lifting, extended skin incision, or others, were performed.

The mean follow-up period after surgery was 13.1 months (range 0.7–34.3 months).

All patients were pleased with avoiding potential needs from previously described techniques, such as two-step surgeries, closed-eye for weeks or damaging further structures. All of the patients were also satisfied with the functional and cosmetic outcomes.

In the early postoperative appointment, mild swelling in three patients (20.0% of cases) and mild ecchymosis in nine patients (60.0% of cases) were the only signs noticed, with no significant complications such as pain, infections, dehiscence of suture or active bleeding.

From their later follow-ups, none (0% of cases) presented with corneal impairment, entropion, ectropion, lagophthalmus, granulomas or flap necrosis. The only symptomatic complication included one patient (6.7% of cases) with trichiasis involving a single eyelash (Section 3.1.15, Patient 15), which required electrolysis. The most frequent finding was mild lid retraction, observed in five patients (33.3% of cases), typically measuring around 0.5 mm, without clinical significance. This could potentially have been prevented by adding some anchoring sutures from the cheek to the periosteum, but this was not performed in any of the patients from this clinical series. Other observations included a minor irregularity of the margin in four patients (Section 3.1.2, Section 3.1.3, Section 3.1.6 and Section 3.1.8 (Patients 2, 3, 6 and 8)) (26.6% of cases), partial madarosis in two patients (Section 3.1.2 and Section 3.1.6 (Patients 2 and 6)) (13.3% of cases), mild flap thickening in two patients (Section 3.1.2 and Section 3.1.5 (Patients 2 and 5)) (13.3% of cases) and a small red area in the margin in one patient (Section 3.1.13, Patient 13) (6.7% of cases), but this situation was observed to settle down with time, and both patients had a short follow-up. Note that the evident difference in the colouration of the flap in Section 3.1.14 (Patient 14) is due to the hypopigmentation of the harvested area, which is unrelated to complications. The results are summarised in Table 1.

### 3.1. Images of Cases

#### 3.1.1. Patient 1

Patient 1 underwent tumour excision and reconstruction without a confirmed diagnosis due to systemic illnesses. The right lower lid was affected by a sebaceous hyperplasia. Following tumour removal, a 70% defect was reconstructed using a remnant lateral lower lid of 25% and an island pedicle flap. There were no complications related to the surgery, though the patient passed away a few months later.

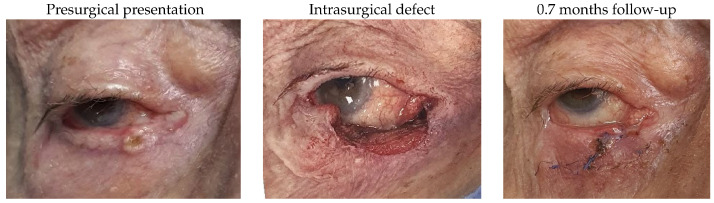



#### 3.1.2. Patient 2

This patient was affected by a right lower lid basal cell carcinoma and presented with a defect of 72% after tumour excision. Reconstruction involved a remnant lateral lower lid of 20% and an island pedicle flap. An asymptomatic flap thickening, irregular margin, 0.3 mm of lid retraction and partial madarosis were identified after surgery. 
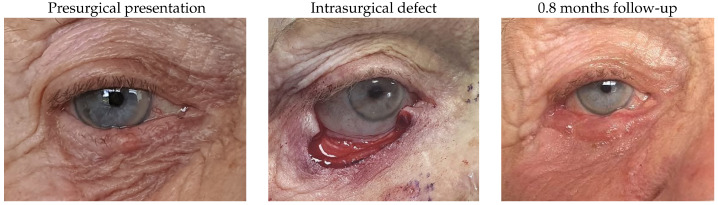


#### 3.1.3. Patient 3

Patient 3 was affected by a right lower lid basal cell carcinoma and underwent tumour excision, resulting in a 50% defect. Reconstruction included a remnant lateral lower lid of 13% and an island pedicle flap. Minimal irregular margin was the only postsurgical complication. 
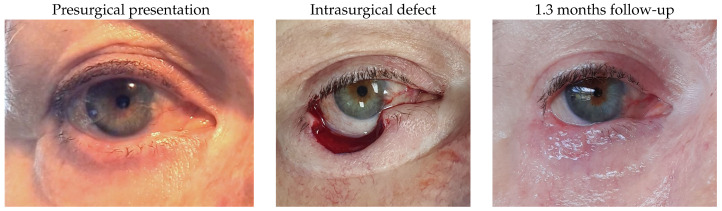


#### 3.1.4. Patient 4

Patient 4 was dealing with a left lower lid basal cell carcinoma and required tumour removal, creating a defect of 77%. Reconstruction was performed using a remnant lateral lower lid of 19% and an island pedicle flap. No complications were observed postoperatively. 
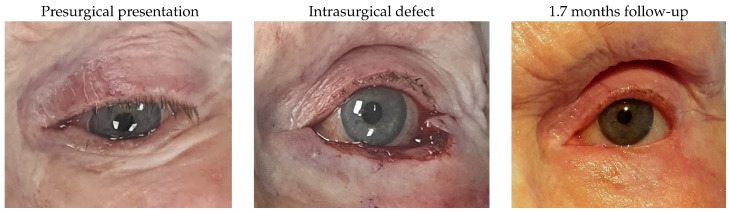


#### 3.1.5. Patient 5

Patient 5, who was affected by a right lower lid basal cell carcinoma, presented with a defect of 79% after tumour excision and underwent canalicular and eyelid reconstruction using a monocanalicular stent, a remnant lateral lower lid of 18% and an island pedicle flap. A flap thickening and 1 mm of lid retraction were complications observed postsurgically. 
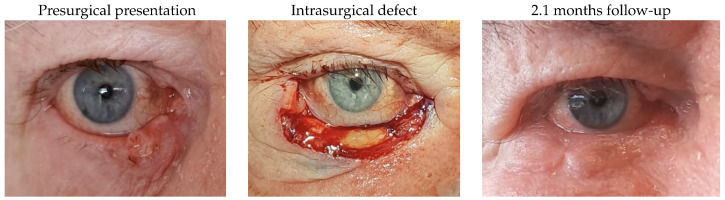


#### 3.1.6. Patient 6

Patient 6 was affected by a right lower lid basal cell carcinoma, leading to a 77% defect after excision. Reconstruction of the lacrimal system and the eyelid involved a monocanalicular stent, a remnant lateral lower lid of 23% and an island pedicle flap. An asymptomatic mild lid irregularity, 1 mm of lid retraction and partial madarosis were documented. 
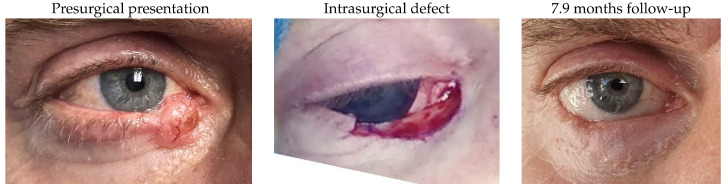


#### 3.1.7. Patient 7

Patient 7 presented with a right lower lid basal cell carcinoma that left a defect of 68% and a remnant lateral lower lid of 29%. The reconstruction included the repair of the inferior canaliculus as well as an island pedicle flap. There were no complications. 
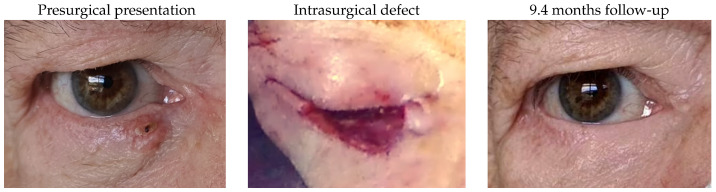


#### 3.1.8. Patient 8

Patient 8, who was affected by a right lower lid basal cell carcinoma, had a defect of 68% after tumour removal and required reconstruction using a remnant lateral lower lid of 14% and an island pedicle flap. An asymptomatic mild irregularity along the margin with 0.5 mm of lid retraction were noted during follow-up. 
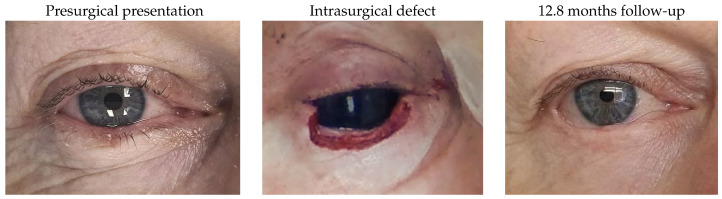


#### 3.1.9. Patient 9

Patient 9, who was affected by a right lower lid basal cell carcinoma, underwent tumour excision with a defect of 68%. The reconstruction included the use of a remnant lateral lower lid of 12% and an advancement flap. No complications were identified. 
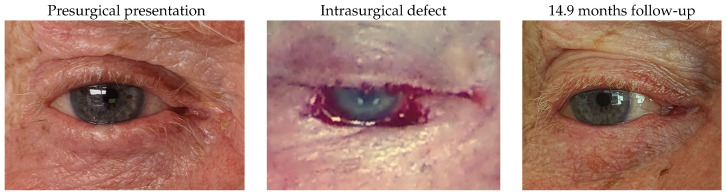


#### 3.1.10. Patient 10

Patient 10, who was affected by a right lower lid basal cell carcinoma, underwent tumour excision with a defect of 62% and reconstruction using a remnant lateral lower lid of 17% and an island pedicle flap. Minimal lid retraction was the only postsurgical complication. 
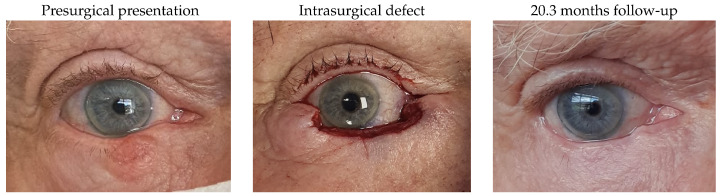


#### 3.1.11. Patient 11

Patient 11, who had a right lower lid basal cell carcinoma, required tumour removal, leaving a defect of 75%. Reconstruction included the use of a remnant lateral lower lid of 14% and an island pedicle flap. The patient experienced no complications. 
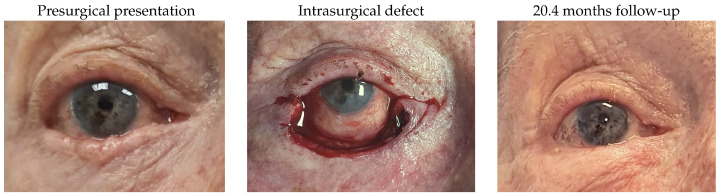


#### 3.1.12. Patient 12

Patient 12, who was affected by a right lower lid basal cell carcinoma, underwent tumour removal and presented a defect of the inferior canaliculus and the inferior eyelid of 79% afterwards. Reconstruction was achieved by the implantation of a Mini-Monoka and the MARCH technique, using a remnant lateral lower lid of 14% and an island pedicle flap. No complications were identified. 
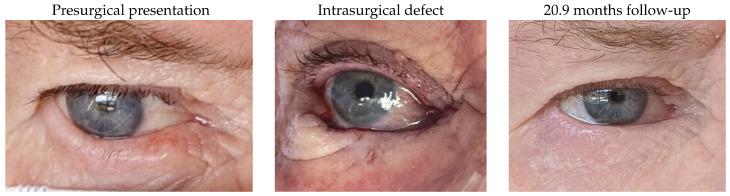


#### 3.1.13. Patient 13

Patient 13 was also affected by a right lower lid basal cell carcinoma. After removal of the tumour, a defect of 58% was reconstructed by this technique, using a remnant lateral lower lid of 23% and an island pedicle flap. A red margin was the only evident complication after surgery. 
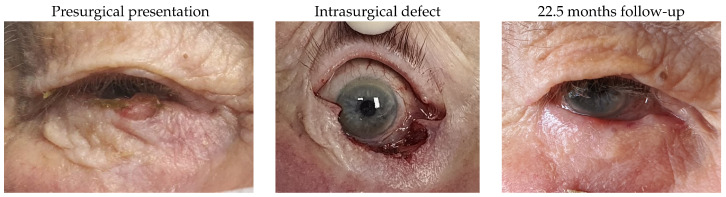


#### 3.1.14. Patient 14

Patient 14, who was affected by a left lower lid basal cell carcinoma, underwent tumour removal, leaving a defect of 68%, and reconstruction using a remnant lateral lower lid of 25% and an island pedicle flap. The evident difference in the colouration of the flap was related to the hypopigmentation of the harvested area. There were no complications. 
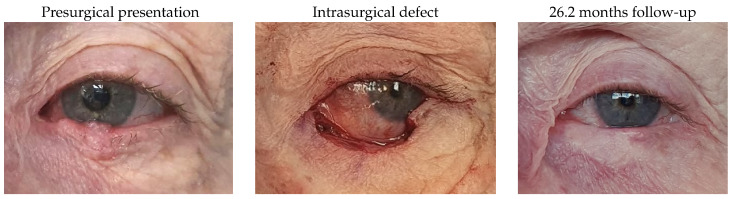


#### 3.1.15. Patient 15

Patient 15 treated with this technique presented with a right lower lid basal cell carcinoma that left a canalicular defect, which required a Mini-Monoka, an eyelid defect of 60% and a remnant lateral lower lid of 37%. Our technique included an island pedicle flap in this case. After surgery, there was a trichiatic eyelash that required one session of electrocautery. 
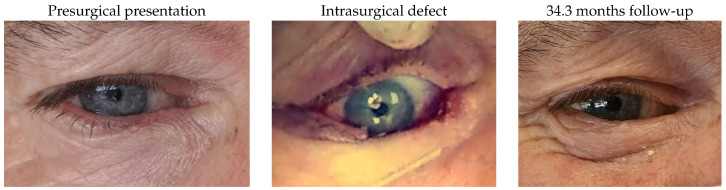


## 4. Discussion

The MARCH technique is a safe surgical alternative for the reconstruction of the lower eyelid in medium- and large-size full-thickness defects up to 79% where there is a remnant lateral lower eyelid.

This technique offers several advantages over other reconstructive methods.

One of the main benefits is that this technique does not sacrifice any other tissue. This is an obvious advantage when avoiding using the upper eyelid’s tarsal plate on the same or the contralateral side, hard palate, periosteum, ear cartilage or nasal septum. However, there are still some less obvious advantages secondary to the minimally invasive use of surrounding tissue, which is, for instance, required in a Tenzel flap, because the sequence in which surgical techniques are employed can significantly influence the feasibility of future interventions. In this regard, starting with the MARCH technique rather than the Tenzel flap better preserves the integrity of surrounding eyelid tissues, including the lateral canthal area. This strategic preservation ensures that, in the event of a recurrence or a new tumour affecting the same lower eyelid, a Tenzel flap can still be performed later. In contrast, if the Tenzel flap is initially used, the mobilisation and redistribution of eyelid tissues inherent to this technique compromise the availability of these same tissues for subsequent procedures, such as the MARCH technique. Thus, employing our procedure as a first-line option provides a critical safeguard for future surgical options, ensuring that a wider range of techniques remains available for managing potential recurrences or new pathologies. Moreover, the minimally aggressive nature also contributes to better tolerance during surgery and better recovery after surgery, as the use of other tissue has potential risks that are avoided in this technique.

Another important advantage is that it involves only a single-stage surgical intervention. To the best of our knowledge, the MARCH technique is the only single-stage approach described for reconstructing lower eyelid defects exceeding 70% of the eyelid length [6,10]. This avoids the need to keep the eye closed for several weeks and eliminates the necessity for a second procedure to open the flaps, as required in the Hughes technique. A single-step surgery has several secondary benefits that include reducing the follow-up visits required, the cost of a second operation, and the time that the patient is on sick leave, which also has an important social and economic cost. All these advantages make the MARCH procedure more convenient and better tolerated by the patients. For the same reasons and because the learning curve is also very easy to achieve for an eyelid surgeon, the operating time is shorter.

The MARCH technique also appears to have a reduced risk of complications. Dry eye, entropion, ectropion, lid retraction, granulomas, trichiasis, dead flaps and grafts, kinking of the tarsus, sagging of the flap, and other complications have been reported with traditional techniques. In our series, we did not observe significant complications. However, we understand that the numbers of this series are low, and we would need bigger numbers to draw firm conclusions regarding the complications rate.

Another interesting point is that this procedure can preserve eyelashes in specific areas to maintain their physiological protection role for the cornea and achieve a better cosmetic result. Nonetheless, it is worth noticing that the eyelashes used are harvested from the remnant lateral side of the eyelid and, therefore, are not as thick, dense and long as the central ones, giving even the gross appearance of madarosis in some cases.

Finally, the fact that both lamellae are managed separately offers an excellent option to reconstruct the lacrimal punctum in a very physiological position.

One of the potential limitations of this technique is the occurrence of mild lid retraction. This complication might be avoided by anchoring additional sutures to the periosteum, slightly lifting the cheek to achieve better lid positioning and avoid inferior pulling during the healing process. However, this approach was not routinely implemented in our series. Further evaluation in a larger series of cases with long-term follow-up is needed to determine whether such modifications would be beneficial for all patients undergoing the MARCH procedure. Moreover, despite the fact that this technique has notably been successfully performed with a remnant lateral lower lid measuring as little as 12% of the total eyelid length, further studies with larger cohorts are required to better define its limitations, considering that its potential may also be influenced by other factors such as defect size and lid laxity. Additionally, despite no significant complications being found, we need to consider that potential ocular surface symptoms and epiphora from functional nasolacrimal duct obstruction may be detected in a larger case series. Nonetheless, we could assume that removing up to nearly 80% of the eyelid may play an important role in it, more than the reconstructive procedure itself.

## 5. Conclusions

The MARCH technique is a single-step surgical intervention for eyelid reconstruction that is minimally invasive and offers optimal results even in extensive defects, up to nearly 80% in certain cases. As an alternative to other surgeries in similar cases, it removes the need for a second operation and preserves other donor tissues, such as the periosteum or tarsus, in anticipation of possible future surgeries. The anatomic, functional and cosmetic results are optimal, with the possibility of guiding the position of the remnant eyelashes and lacrimal system’s tube in full-thickness medial and central lower eyelid defects. The MARCH technique is an excellent first-line approach for eyelid reconstruction.

## Figures and Tables

**Table 1:** Demographics and characteristics of patient tumour, lid defect, surgery and outcomes following reconstruction with the MARCH technique.

Patients:	1	2	3	4	5	6	7	8	9	10	11	12	13	14	15
Age (years)	95	86	76	88	83	53	74	48	81	65	86	67	82	70	55
Anaesthetics	LA	LA	LA	LA	LA	GA	S	LA	LA	LA	LA	LA	LA	LA	LA
Involved lid	RLL	RLL	RLL	LLL	RLL	RLL	RLL	RLL	RLL	RLL	RLL	RLL	RLL	LLL	RLL
Diagnosis	SH	BCC	BCC	BCC	BCC	BCC	BCC	BCC	BCC	BCC	BCC	BCC	BCC	BCC	BCC
Defect size (mm)	17	18	15	20	22	23	19	19	17	18	21	22	15	19	18
Defect size (%)	70	72	50	77	79	77	68	68	68	62	75	79	58	68	60
RLLL size (mm)	6	5	4	5	5	7	8	4	3	5	4	4	6	7	11
RLLL size (%)	25	20	13	19	18	23	29	14	12	17	14	14	23	25	37
Operative time (minutes)	61	36	60	55	60	50	69	82	38	60	35	78	67	60	52
LS repair	no	no	no	no	yes	yes	yes	no	no	no	no	yes	no	no	yes
Follow-up (months)	0.7	0.8	1.3	1.7	2.1	7.9	9.4	12.8	14.9	20.3	20.4	20.9	22.5	26.2	34.3
Complications	-	FT, IM,	IM	-	FT, LR	IM, LR,	-	LR	-	LR	-	-	RA	-	T
		LR, PM				PM									

BCC, basal cell carcinoma. F, female. FT, flap thickening. GA, general anaesthesia. IM, irregularity of the margin. LA, local anaesthesia. LLL, left lower lid. LR, lid retraction. LS, lacrimal system. M, male. PM, partial madarosis. RA, red area. RLL, right lower lid. RLLL, Remnant lateral lower lid. S, sedation. SH, sebaceous hyperplasia. T, trichiasis.

## Data Availability

Data is unavailable due to privacy and ethical restrictions.
